# Supplementary figures and images for: Water, sanitation, and hygiene (WASH) factors and the incidence of communicable diseases in Urban Bangladesh: Evidence from municipal areas
Source: PLoS Negl Trop Dis. 2025 Jul 31;19(7):e0013329. doi: 10.1371/journal.pntd.0013329 (PMC12312932; doi:10.1371/journal.pntd.0013329)

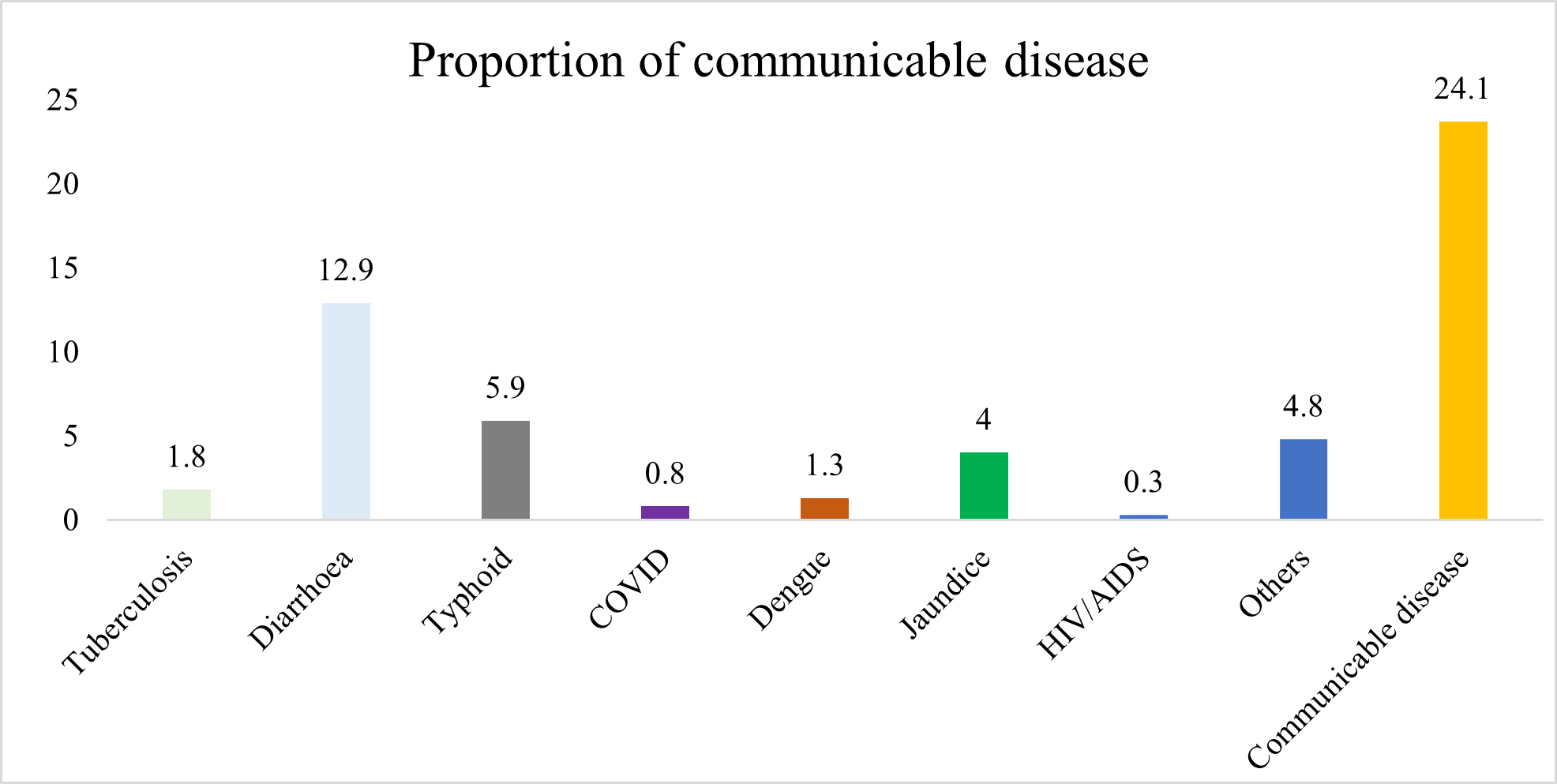

Supplement: S1 Fig — This figure shows the distribution of self-reported communicable diseases among affected participants. Diarrhea was the most reported illness, followed by typhoid, jaundice, tuberculosis, dengue, COVID-19, and HIV/AIDS. (TIF) [file pntd.0013329.s001.tif]
