# Supplementary material for: Water, sanitation, and hygiene (WASH) factors and the incidence of communicable diseases in Urban Bangladesh: Evidence from municipal areas
Source: PLoS Negl Trop Dis. 2025 Jul 31;19(7):e0013329. doi: 10.1371/journal.pntd.0013329 (PMC12312932; doi:10.1371/journal.pntd.0013329)
Supplement: S1 Table — A. Socio-demographic characteristics of the study participants (n = 607). This table presents participants’ age, gender, religion, education, occupation, marital status, income, and housing type. B. Hygiene-related and disease-related variables (n = 607). Details participants’ water source, toilet type, handwashing practices, garbage disposal methods, chronic and communicable disease history, and use of the PEPSEP voucher program. C. Bivariate associations between sociodemographic and hygiene-related variables and communicable disease status (n = 607). Displays chi-square test results indicating significant and non-significant associations between selected variables and communicable disease occurrence. D. Multivariable logistic regression analysis identifying predictors of communicable diseases (n = 607). Presents adjusted odds ratios and confidence intervals for key variables such as gender, toilet type, water source, handwashing, and chronic illness. (DOCX) [file pntd.0013329.s002.docx]

| ***Table A. Socio-demographic profile of the respondents (n = 607)*** | | |
| --- | --- | --- |
| **Variable** | **Categories** | ***n (%)*** |
| **Age(years)** | ≤ 30  31-40  41-50  51 ≥ | 246 (40.5)  223 (36.7)  76 (12.5)  62 (10.2) |
| **Gender** | Male  Female | 99 (16.3)  508 (83.7) |
| **Religion** | Muslim  Hindu | 530 (87.3)  77 (12.7) |
| **Education level** | No education  PSC  JSC  SSC and above | 140 (23.1)  314 (51.7)  100 (16.5)  53 (8.7) |
| **Employment status** | Housewife  Service holder (Gov & private, garment worker)  Labourer  Others | 384 (63.3)  63 (10.4)  113 (18.6)  47 (7.7) |
| **Marital status** | Married  Widowed/Separated/Single/Divorced | 572 (94.2)  35 (5.8) |
| **Family income** | ≤ 5000  5001-10000  10001-15000  ≥15001 | 64 (10.5)  303 (49.9)  193 (31.8)  47 (7.7) |
| **Household type** | Raw (Shack, Mud)  Tin shed  Semi building  Building | 91 (15.0)  208 (34.3)  253 (41.7)  55 (9.1) |
|  | | |

| ***Table B. Hygiene-related and disease factors of the study participants (n = 607)*** | | |
| --- | --- | --- |
| **Variable** | **Categories** | ***n (%)*** |
| **Source of drinking water** | Tube well  Others | 535 (88.1)  72 (11.9) |
| **Type of toilet** | Sanitary latrine  Ring slab with water seal | 401 (66.1)  206 (33.9) |
| **Hand washing practice (before or after food)** | Yes  No | 574 (94.6)  33(5.4) |
| **Hand washing practice (before or after toilet)** | Yes  No | 580 (95.6)  27 (4.4) |
| **Garbage dump** | Household dustbin/collected by municipality person  Others | 284 (46.8)  323 (53.2) |
| **History of chronic disease** | Yes  No | 260 (42.8)  347 (57.2) |
| **Type of chronic disease** | Hypertension  Diabetes  Heart disease  Cancer  Stroke  CKD  Asthma | 150 (24.7)  146 (24.1)  30 (4.9)  1 (0.2)  10 (1.6)  15 (2.5)  37 (6.1) |
| **History of communicable disease** | Yes  No | 146 (24.1)  461 (75.9) |
| **Regular visits of PEPSEP volunteers** | Yes  No | 605 (99.7)  2 (0.3) |
| **Adequacy of PEPSEP voucher** | Yes  No | 479 (78.9)  128 (21.1) |

| ***Table C. Socioeconomic factors and their association with communicable disease (n=607)*** | | | | |
| --- | --- | --- | --- | --- |
| **Table** | **Communicable disease** | | **Total** | ***p-value*** |
|  | **Yes** | **No** |  |  |
| **Age (In years)**  ≤30  31-40  41-50  51 ≥ | 61 (24.8)  49 (22.0)  17 (22.4)  19 (30.6) | 185 (75.2)  174 (78.0)  59 (77.6)  43 (69.4) | 246 (40.5)  223 (36.7)  76 (12.5)  62 (10.2) | 0.533 |
| **Gender**  Male  Female | 17 (17.2)  129 (25.4) | 82 (82.8)  379 (74.6) | 99 (16.3)  508 (83.7) | 0.080 |
| **Education level**  No education  PSC  JSC  SSC and above | 35 (25.0)  65 (20.7)  30 (30.0)  16 (30.2) | 105 (75.0)  249 (79.3)  70 (70.0)  37 (76.3) | 140 (23.1)  314 (51.7)  100 (16.5)  53 (8.7) | 0.170 |
| **Employment status**  Housewife  Service holder  Laborer  Others | 94 (24.5)  17 (27.0)  26 (23.0)  9 (19.1) | 290 (75.5)  46 (73.0)  87 (77.0)  38 (80.9) | 384 (63.3)  63 (10.4)  113 (18.6)  47 (7.7) | 0.796 |
| **Marital status**  Married  Widowed/Separated/Single/Divorced | 141 (24.7)  5 (14.3) | 431 (75.3)  30 (85.7) | 572 (94.2)  35 (5.8) | 0.164 |
| **Family income**  ≤5000  5001-10000  10001-15000  15001 ≥ | 6 (9.4)  68 (22.4)  55 (28.5)  17 (36.2) | 58 (90.6)  235 (77.6)  138 (71.5)  30 (63.8) | 64 (10.5)  303 (49.9)  193 (31.8)  47 (7.7) | **0.003** |
| **Household type**  Raw (Shack, Mud)  Tin shed  Semi building  Building | 30 (33.0)  46 (22.1)  58 (22.9)  12 (21.8) | 61 (67.0)  162 (77.9)  195 (77.1)  43 (78.2) | 91 (15)  208 (34.3)  253 (41.7)  55 (9.1) | 0.194 |
| **Regular visits of PEPSEP volunteers**  Yes  No | 146 (24.1)  0 (0.0) | 459 (75.9)  2 (100.0) | 605 (99.7)  2 (0.3) | 0.425 |
| **Adequacy of PEPSEP voucher**  Yes  No | 109 (22.8)  37 (28.9) | 370 (77.2)  91 (71.1) | 479 (78.9)  128 (21.1) | 0.148 |
| **History of chronic disease**  Yes  No | 93 (35.8)  53 (15.3) | 167 (64.2)  294 (84.7) | 260 (42.8)  347 (57.2) | **<0.001** |
| **Source of drinking water**  Tube well  Others | 117 (21.9)  29 (40.3) | 418 (78.1)  43 (59.7) | 535 (88.1)  72 (11.9) | **0.001** |
| **Type of toilet**  Ring slab with water seal Sanitary latrine | 62 (30.1)  84 (20.9) | 144 (69.9)  317 (79.1) | 206 (33.9)  401 (66.1) | **0.013** |
| **Hand washing practice (before or after food)**  Yes  No | 117 (20.4)  29 (87.9) | 457 (79.6)  4 (12.1) | 574 (94.6)  33 (5.4) | **<0.001** |
| **Hand washing practice (before or after toilet)**  Yes  No | 122 (21.0)  24 (88.9) | 458 (79.0)  3 (11.1) | 580 (95.6)  27 (4.4) | **<0.001** |
| **Garbage dump**  Household dustbin/collected by municipality person  Others | 48 (16.9)  98 (30.3) | 236 (83.1)  225 (69.7) | 284 (46.8)  323 (53.2) | **<0.001** |

***Table D. Association of different communicable diseases with explanatory factors***

***identified from logistic regression***

| Variable | | ***AOR*** | ***95% CI*** | ***P- value*** |
| --- | --- | --- | --- | --- |
| Age category | 30 or below(ref) |  |  |  |
|  | 31-40 | 2.06 | 0.94to 4.51 | 0.07 |
|  | 41-50 | 2.48 | 0.98 to 5.82 | 0.112 |
|  | 51 or more | 4.67 | 0.85 to 11.3 | 0.20 |
| Gender | Female | 3.21 | 1.19 to 8.66 | 0.003 |
|  | Male (ref) |  |  |  |
| Occupation | Housewife | 1.81 | 0.52 to 2.56 | 0.72 |
|  | Service holder | 2.07 | 0.58 to 2.76 | 0.56 |
|  | Laborer | 1.56 | 0.94 to 4.81 | 0.65 |
|  | Others (ref) |  |  |  |
| Education | Illiterate(ref) |  |  |  |
|  | PSC | 1.75 | 0.32 to 1.57 | 0.44 |
|  | JSC | 2.02 | 0.42 to 2.05 | 0.25 |
|  | SSC and above | 2.46 | 0.74 to 2.98 | 0.19 |
| Marital status | Married | 2.14 | 0.87 to 5.65 | 0.65 |
|  | Single (ref) |  |  |  |
| Income | 5000 or below(ref) |  |  |  |
|  | 5001-10000 | 2.94 | 0.62 to 3.28 | 0.23 |
|  | 10001-15000 | 3.21 | 0.78 to 3.76 | 0.19 |
|  | 15001 or more | 1.18 | 0.55 to 1.57 | 0.32 |
| Household type | Raw(ref) |  |  |  |
|  | Tin shed | 1.54 | 0.72 to 2.19 | 0.27 |
|  | Semi building | 2.09 | 0.65 to 4.34 | 0.45 |
|  | Building | 1.76 | 0.89 to 2.76 | 0.49 |
| Source of drinking water | Tube well | 2.81 | 1.13 to 7.02 | 0.015 |
|  | Others (ref) |  |  |  |
| Type of toilet | Sanitary latrine | 0.18 | 0.07 to 0.49 | 0.002 |
|  | Ring slab with water seal (ref) |  |  |  |
| Hand washing before or after eating | Yes (ref) |  |  |  |
|  | No | 23.1 | 1.98 to 69.41 | 0.003 |
| Hand washing before or after using toilet | Yes (Ref) |  |  |  |
|  | No | 12.31 | 2.86 to 53.03 | 0.000 |
| Household garbage dumping | Household dustbin/collected by municipals | 3.47 | 0.98 to 4.78 | 0.154 |
|  | Others (ref) |  |  |  |
| History of chronic disease | Yes | 3.73 | 1.49 to 9.59 | 0.007 |
|  | No (ref) |  |  |  |

*AOR = Odds ratio (Adjusted), CI= Confidence interval, ref= reference categories, P value <0.05 were found to be statistically significant.*
