# Supplementary material for: Water, sanitation, and hygiene (WASH) factors and the incidence of communicable diseases in Urban Bangladesh: Evidence from municipal areas
Source: PLoS Negl Trop Dis. 2025 Jul 31;19(7):e0013329. doi: 10.1371/journal.pntd.0013329 (PMC12312932; doi:10.1371/journal.pntd.0013329)
Supplement: S1 Questionnaire — The structured questionnaire was developed based on WHO’s Core Questions on WASH for Household Surveys and translated into Bengali. (PDF) [file pntd.0013329.s003.pdf]

DHAKA AHSANIA MISSION (DAM)
HEALTH SECTOR
152/Ka PC Culture Housing, Shyamoli, Dhaka 1207

&

CHRISTAIN AID

***Supported by*: The EUROPEAN UNION**

পেপসেপ প্রকল্পের এন্ড লাইন জরিপ এবং মূল্যায়ন পরিচালনার প্রশ্নপত্র

Area ID		

Participant ID				

সম্মতিপত্র
(সাক্ষাৎকার প্রদানকারীর সাক্ষাৎকারে অংশগ্রহণের সম্মতিপত্র)

আস্‌সালামু-ওয়ালাইকুম। আমি ....ঢাকা আহ্‌ছানিয়া মিশনের স্বাস্থ্য বিভাগ ও খ্রিস্টান এইড কর্তৃক বাস্তবায়িত "পেপসেপ প্রকল্পের এন্ড লাইন জরিপ এবং মূল্যায়ন পরিচালনা করছি" প্রকল্পে কর্মরত একজন তথ্য/উপাত্ত সংগ্রহকারী। আমি আপনাকে এই জরিপে অংশগ্রহনেরআমন্ত্রন জানাচ্ছি। এই জরিপে আপনার অংশগ্রহন সম্পূর্ণ ঐচ্ছিক এবং আপনি চাইলেই যেকোন প্রশ্নের উত্তর এড়িয়ে যেতে পারেন।আপনি যদি এই জরিপে অংশগ্রহন না করেন, অথবা কোন অংশ উত্তর না দেয়ার সিদ্ধান্ত নেন, তাহলেও তা আপনার সাথেআমাদের ভবিষ্যত সম্পর্কে কোন প্রভাব ফেলবে না। আপনি অংশগ্রহনের সিদ্ধান্ত নিলেও যেকোন সময় এটা থেকে নাম প্রত্যাহার করে নিতে পারেন। আপনার যদি কোন প্রশ্ন থাকে তাহলে তা এখন জিজ্ঞেস করতে পারেন। এই জরিপে অংশগ্রহনে আপনার কোনঝুঁকি নেই, এবং এ থেকে আপনি তাৎক্ষণিক কোন সুবিধাও পাবেন না। আপনার থেকে প্রাপ্ত তথ্য পেপসেপ প্রকল্প বাস্তবায়ন পরিবর্তী গবেষণায় উল্লেখযোগ্যভাবে সহযোগিতা করবে। আপনার থেকে প্রাপ্ত তথ্য অত্যন্তগোপনীয়ভাবে রাখা হবে এবং এই তথ্যগুলো থেকে ভবিষ্যতে আপনাকে কেউ খুঁজে বের করতে পারবে না।
সম্মতির বিবৃতি: আমি উপরে উল্লেখিত তথ্য বুঝেছি এবং আমি আমার প্রশ্নের উত্তর পেয়েছি। আমি এই জরিপে অংশ নিতে সম্মত।

এই গবেষণা সম্পর্কে যদি আপনার কোন প্রশ্ন থাকে তাহলে আপনি জনাব মোঃ মোখলেছুর রহমান প্রকল্প ব্যবস্থাপক, স্বাস্থ্য বিভাগ, ঢাকা আহ্‌ছানিয়া মিশন, ১৫২/ক, পিসিকালচার হাউজিং সোসাইটি, শ্যামলী, ঢাকা-১২০৭ (টেলিফোন নংঃ ০২-৫-৮১৫১১১৪) এর সাথে যোগাযোগ করতে পারেন।

চলমান এই গবেষণা কাজে বর্তমানে এবং প্রয়োজনে পরবর্তীতে তথ্য/উপাত্ত দিয়ে সহায়তা করতে আপনি কি সম্মত আছেন? (নীচের বক্সে ✓টিক চিহ্ন দিন)।

☐হ্যাঁ ☐না

স্বাক্ষর/টিপসই ---------------

জরিপকাজেঅংশগ্রহনকারীরপরিচিতি ঃ

অংশগ্রহনকারীর উপজেলা	

01:সাভার; **02:** সাতক্ষীরা

সাক্ষাৎকার গ্রহনকারীর নাম ঃ ------------------------------------- তারিখ ঃ --------------------

A. উত্তরদাতা ও খানার সাধারণ তথ্য ঃ (খানা বলতে একই হাড়িতে রান্না করে খায় এমন সদস্যদের বুঝানো হয়েছে) ঃ

101	অংশগ্রহনকারীর তথ্য (কোড লিখতে হবে)				
a. লিঙ্গ	b. বয়স (in year)	c. পেশা	d. শিক্ষা	e. বৈবাহিক অবস্থা	f. ধর্ম

লিঙ্গ ঃ	১ = নারী , ২ = পুরুষ, ৩ = তৃতীয় লিঙ্গ (হিজড়া)
পেশা ঃ	১= গৃহিনী, ২= সরকারী চাকুরী, ৩= বেসরকারী চাকুরী, ৪= শ্রমিক, ৫= পোশাক শ্রমিক, ৬= ক্ষুদ্র ব্যবসায়ী/ ফেরিওয়ালা, ৭= কৃষিজীবি, ৮ =মিস্ত্রী, ৯ = দিন-মজুর, ১০ = চা বিক্রেতা, ১১= ড্রাইভার, ১২= রিকসা/ভ্যান/ইজিবাইক চালক, ১৩= অন্যান্য
শিক্ষা ঃ	১=নিরক্ষর, ২=প্রাথমিক/সমমান, ৩= অষ্টম/সমমান, ৪=মাধ্যমিক/সমমান, ৫= উচ্চ মাধ্যমিক/সমমান, ৬= স্নাতক/তার উপরে/ সমমান
বৈবাহিক অবস্থা ঃ	১ = অবিবাহিত, ২ = বিবাহিত, ৩ =বিধবা/ বিপত্নীক, ৪ = তালাক প্রাপ্ত
ধর্ম ঃ	১ = মুসলিম, ২ = হিন্দু ৩ = খ্রীস্টান, ৪ = বৌদ্ধ, ৫ = অন্যান্য - - - - - - - - - - - - - -- - - - - - - - - -

B. আর্থ-সামাজিক তথ্য (Socioeconomic Information)

201	খানার মাসিক মোট আয় কত?		
202	খানার মাসিক মোট খরচ/ব্যয় কত?		
203	খানার বাস গৃহের ধরণ ঃ	১= ঝুঁপড়ি, ২ = কাঁচা, ৩= টিনসেড, ৪ = সেমি পাকা, ৫= পাকা	
204	বাস গৃহের মালিকানা ঃ	১=নিজস্ব, ২=ভাড়া, ৩ = অন্যের জমিতে বসবাস, ৪. অন্যান্য---------------	
205	অস্থায়ী বাসিন্দা বা ভাড়াটিয়া হলে পরিবারটির নিজ জেলা/দেশের বাড়ী কোথায় ?		
206	আপনি এই এলাকায় কত বছর ধরে বসবাস করছেন?		- - - - - - - - - - - - - বছর
207	আপনার গৃহে বিদ্যুৎ সংযোগ আছে কিনা ঃ	১= হ্যাঁ ২= না	

208			
পারিবারিক সম্পদ ঃ<br>ডান পার্শ্বের কলামে উল্লেখিত বিষয়গুলির কোনটি আপনার আছে?<br>(একাধিক উত্তর গ্রহনযোগ্য)<br>গোল (O) চিহ্ন দিয়ে সনাক্ত করুণ।	(১) গরু<br>(২) ছাগল<br>(৩) রিক্সা/ ভ্যান<br>(৪) ইজি বাইক<br>(৫) বাই সাইকেল<br>(৬) মোটর সাইকেল<br>(৭) ফ্রিজ	(৮) টেলিভিশন<br>(৯) মোবাইল<br>(১০) অ্যান্ড্রেয়ড মোবাইল<br>(১১) ইলেকট্রিক ফ্যান<br>(১২) সেলাই মেশিন<br>(১৩) কম্পিউটার<br>(১৪) ল্যাপটপ	(১৫) খাট/চৌকি<br>(১৬) শোকেস<br>(১৭) টেবিল<br>(১৮) চেয়ার<br>(১৯) অন্যান্য (নির্দিষ্ট করুন) - - - - - - - - - - -

209	আপনার খানার খাবার পানির প্রধান উৎস কি?	১) পৌরসভার সরবরাহকৃত পানি; ২) গভীর নলকুপ; ৩) নলকুপ;<br>৪) অন্যান্য (নির্দিষ্ট করুন) - - - - - - - -	
210	আপনার বাড়িতে কি ধরনের বাথরুম/টয়লেট আছে?	১) নর্দমার সঙ্গে সংযুক্ত ল্যাট্রিন; ২) সেপ্টিক ট্যাংকের সঙ্গে সংযুক্ত স্যানিটারি ল্যাট্রিন; ৩) রিং-স্লাব ওয়াটার সিল সহ; ৪) ঝুলন্ত/কাঁচা টয়লেট; ৫) অন্যান্য	
211	আপনি রান্নার জন্য প্রধানতঃ কোন ধরনের জ্বালানী ব্যবহার করেন ?	১) বিদ্যুৎ (ইন্ডাকশন চুলা/রাইস কুকার); ২) সিলিন্ডার গ্যাস; ৩) সাপ্লাই গ্যাস (তিতাস);<br>৪) বায়োগ্যাস; ৫) কাঠ; ৬) অন্যান্য - - - - - - - - - - - - - - -	
212	আপনি এবং আপনার পরিবারের সবাই খাবার আগে ও পরে কি হাত ধৌত করেন?	১= হ্যাঁ ২= না	
213	আপনি এবং আপনার পরিবারের সবাই কি বাথরুম/টয়লেট ব্যবহারের পরে সাবান/ ডিটারজেন্ট/অন্যান্য দ্রব্য দিয়ে হাত ধৌত করেন?	১= হ্যাঁ ২= না	
214	বাসা/র বর্জ্য পদার্থ আপনি কি করেন ?	১) বাসার ডাস্টবিনে ফেলেন, পৌরসভার লোক নিয়ে যায়; ২) খোলা জায়গায় ফেলে দেন; ৩) এলাকায় অবস্থিত পৌরসভার ডাস্টবিনে ফেলে দেন; ৪) অন্যান্য (নির্দিষ্ট করুন)	

C. সাধারণ ও অন্যান্য চিকিৎসা সংক্রান্ত তথ্য ঃ

301	আপনার এলাকার মধ্যে নিকটস্থ কোন স্বাস্থ্যকেন্দ্র/হাসপাতাল আছে কি?	১= হ্যাঁ ২= না	
302	আপনি কি জানেন আপনার এলাকায় কোথাও বিনামূল্যে চিকিৎসা ব্যবস্থা আছে কি না ?	১= হ্যাঁ ২= না	
303	স্বাস্থ্যসেবা কার্যক্রম থেকে কোন স্বাস্থ্যকর্মী/ (পেপসেপ স্বাস্থ্যকর্মী) আপনার এলাকায় নিয়মিত আসতো কি ?	১= হ্যাঁ ২= না	
304	পেপসেপ থেকে স্বাস্থ্য সেবার জন্য যে টাকা পেয়েছিলেন তা আপনার পরিবারের স্বাস্থ্যসেবার জন্য যথেষ্ট ছিল কি?	১= হ্যাঁ ২= না	
305	স্বাস্থ্যসেবা নেওয়ার জন্য কোথায় গিয়েছিলেন / আপনারা কোথায় যান? (একাধিক উত্তর গ্রহনযোগ্য)	১= সরকারী হাসপাতাল, ২= এনজিও পরিচালিত ক্লিনিক, ৩= প্রাইভেট ক্লিনিক, ৪= প্রাইভেট ডাক্তার, ৫= হোমিওপ্যাথিক ডাক্তার, ৬= স্থানীয় ফার্মেসী, ৭= অন্যান্য	
306	সর্বশেষ স্বাস্থ্যসেবা নেওয়ার জন্য কত টাকা খরচ হয়েছিল?		

**307**	আপনার পরিবারে গত সারে তিনবছরে মোট কতজন শিশু জন্মগ্রহণ করেছে?		
**308**	আপনার পরিবারের কতজন সদস্য গত সারে তিন বছরে মারা গেছেন?		
**309**	আপনার পরিবারের গত সারে তিন বছরে পাঁচ বছরের কম বয়সী শিশু মারা গেছেন?		
**310**	গত সারে ৩ বছরে আপনার পরিবারে কত জন্য মায়ের মৃত্যু (গর্ভকালীন, প্রসবের সময় বা প্রসব পরবর্তী দেড় মাসের মধ্যে) হয়েছে?		
**311**	শিশুর ডেলিভারি (জন্ম) কোথায় হয়েছিল?	১= বাসা, ২= হাসপাতাল, ৩. প্রযোজ্য নয়	
**312**	কি ধরণের ডেলিভারি হয়েছিল?	১= স্বাভাবিক, ২= সি-সেকশন, ৩. প্রযোজ্য নয়	
**313**	গর্ভকালীন সময়ে আপনি/আপনার পরিবারের কেউ দক্ষ সাস্থ্যকর্মীর কাছ থেকে স্বাস্থ্যসেবা নিয়েছিলেন কি?	১= হ্যাঁ ২= না	
**314**	গর্ভকালীন সময়ে স্বাস্থ্যসেবা নিয়ে থাকলে, ৪ বার বা তার বেশী নেয়া হয়েছিল কিনা?	১= হ্যাঁ, ২= না, ৩= প্রযোজ্য নয়	
**315**	প্রসবজনিত জটিলতার ক্ষেত্রে আপনি/আপনার পরিবারের কেউ স্বাস্থ্যকেন্দ্র বা দক্ষ স্বাস্থ্যকর্মীর সেবা পেয়েছিলেন কিনা?	১= হ্যাঁ, ২= না, ৩= প্রযোজ্য নয়	
**316**	শিশু জন্মের ২ দিনের মধ্যে মা এবং শিশুর স্বাস্থ্য কোন স্বাস্থ্যকেন্দ্র বা দক্ষ স্বাস্থ্যকর্মী দিয়ে পরীক্ষা করা হয়েছিল কিনা?	১= হ্যাঁ , ২= না, ৩= প্রযোজ্য নয়	
**317**	জন্মের সময় শিশুর ওজন কি স্বাভাবিকের চেয়ে কম ছিল?	১= হ্যাঁ, ২= না, ৩= প্রযোজ্য নয়	
**318**	গত তিন বছরে আপনার বা আপনার পরিবারের কারো গর্ভপাত হয়েছিল কিনা?	১= হ্যাঁ, ২= না, ৩= প্রযোজ্য নয়	
**319**	গর্ভপাত পরবর্তী সময়ে কোন স্বাস্থ্যসেবা কেন্দ্র বা দক্ষ স্বাস্থ্যকর্মী থেকে চিকিৎসা সেবা নেয়া হয়েছিল কিনা?	১= হ্যাঁ, ২= না, ৩= প্রযোজ্য নয়	
**320**	**২ বছরের কম বয়সী শিশুর টিকাদান সংক্রান্ত তথ্য (Vaccination information for children less than 2 years)**		
	এই অংশটি শুধুমাত্র ২ বছরের কম বয়সী শিশুদের টিকাদানের তথ্য নেয়ার জন্য। ২ বছরের কম বয়সী কোন শিশু না থাকলে পরের মডিউলে যান।		
	a. আপনি কি আপনার এই শিশুকে তার বয়স অনুযায়ী সবগুলিটিকা দিয়েছেন? (১= হ্যাঁ, ২= না)		
	b. আপনার এই শিশুর কি টিকার কার্ড আছে? (১= হ্যাঁ, ২= না)		
	c. টিকার কার্ড দেখে লিপিবদ্ধ করুন, নিচের টিকাগুলো দেয়া আছে কিনা? *পাশের ঘরে কোর্ড নম্বর লিখুন।* (১= হ্যাঁ, ২= না)	১) বি সি জি<br>২) পোলিও<br>৩) পেন্টাভ্যালেন্ট	৪) হাম/এম আর<br>৫) পি সি ভি
**321**	আপনার বা আপনার পরিবারের কোন শিশু (৬-৫৯ মাস) ভিটামিন এ ক্যাপসুল খেয়েছিল কিনা?	১= হ্যাঁ, ২= না	
**322**	নবজাতক সব প্রয়োজনীয় স্বাস্থ্যসেবা পেয়েছিল কিনা?	১= হ্যাঁ, ২= না	
**323**	আপনার পরিবারের কিশোর/কিশোরীরা স্বাস্থ্যসেবা সংক্রান্ত তথ্য (মাসিক কখন শুরু হয়, মাসিক চক্র, কৈশোরে গর্ভধারণের কুফল, বাল্য বিবাহের কুফল) পেয়েছিল কিনা?	১= হ্যাঁ, ২= না	
**324**	পেয়ে থাকলে কার কাছ থেকে পেয়েছিল?	১= স্বাস্থ্যসেবা কেন্দ্র, ২= পেপেসেপ স্বেচ্ছাসেবী, ৩. অন্যান্য স্বেচ্ছাসেবী	
**325**	আপনার বা আপনার পরিবারের সদস্যরা পরিবার পরিকল্পনা গ্রহণ করার জন্য কারো পরামর্শ নিয়েছিলেন কিনা?	১= হ্যাঁ, ২= না	
**326**	পরিবার পরিকল্পনা সংক্রান্ত পরামর্শ নিয়ে থাকলে তা কার কাছ থেকে নিয়েছিলেন? (একাধিক উত্তর গ্রহনযোগ্য)	১= স্বাস্থ্যসেবা কেন্দ্র, ২= পেপেসেপ স্বেচ্ছাসেবী, ৩= পরিবারের কোন সদস্য, ৪= অন্যান্য স্বেচ্ছাসেবী	
**327**	আপনার বা আপনার পরিবারের সদস্যরা পরিবার পরিকল্পনার কোন পদ্ধতি গ্রহণ করেছেন কিনা?	১= হ্যাঁ, ২= না	
**328**	যদি করা হয়ে থাকে, সেটি কোন পদ্ধতি? (একাধিক উত্তর গ্রহনযোগ্য)	১= সাময়িক (a. কনডম, b. গর্ভনিরোধক, c. Pill, d. Injection, e. Norplant, f. IUD, g. Natural Method), ২ = স্থায়ী (a. Vasectomy, b. Tubectomy) ৩. Others (Specify)	
**329**	এ পদ্ধতি গুলো আপনার এলাকায় সহজলভ্য কিনা?	১= হ্যাঁ, ২= না	

D. মডিউল: (মা ও শিশুর স্বাস্থ্য ও পুষ্টি)

**401**	শিশু- কে শুধু ৬ মাস পর্যন্ত বুকের দুধ দেয়া হয়েছিল কিনা? (১= হ্যাঁ, ২= না)	
**402**	৬ মাসের পর থেকে শিশু কে বুকের দুধের পাশাপাশি অন্য স্বাভাবিক খাবার দেয়া হয়েছিলেন কিনা? (১= হ্যাঁ, ২= না)	
**403**	শিশুর নিয়মিত স্বাস্থ্য পরীক্ষা (ওজন, উচ্চতা) করা হয়েছিল কিনা? (১= হ্যাঁ, ২= না)	
**404**	গর্ভকালীন সময়ে মা নিয়মিত পুষ্টিকর খাবার খেতেন কিনা? (১= হ্যাঁ, ২= না)	
**405**	গর্ভকালীন সময়ে মা প্রতিদিন তিন বেলা স্বাভাবিক খাবার খেয়েছিল কিনা? (১= হ্যাঁ, ২= না)	

**E. মডিউল: (রোগ সংক্রান্ত তথ্যাবলী)**			
**501**	আপনার বা আপনার পরিবারের কারো গত এক বছরে কোন সংক্রামক রোগ হয়েছিল কিনা? (১= হ্যাঁ, ২= না)		
**502**	যদি থাকে, কোন ধরণের সংক্রামক রোগ আছে? (একাধিক উত্তর গ্রহনযোগ্য)	১= কোভিড-১৯, ২= যক্ষ্মা, ৩= ডায়রিয়া, ৪= টাইফয়েড, ৫= ডেঙ্গু, ৬= জন্ডিস, ৭=এইচআইভি/এইডস, ৮= অন্যান্য	
**503**	সংক্রামক রোগের জন্য কোন জায়গা থেকে স্বাস্থ্য সেবা নেয়া হয়েছিল?	১= সরকারী হাসপাতাল, ২= এনজিও পরিচালিত ক্লিনিক, ৩= প্রাইভেট ক্লিনিক, ৪= প্রাইভেট ডাক্তার, ৫= হোমিওপ্যাথিক ডাক্তার, ৬= স্থানীয় ফার্মেসী, ৭= অন্যান্য	
**504**	আপনার বা আপনার পরিবারের কোন সদস্যকে সংক্রামক রোগের উন্নত চিকিৎসার জন্য কোন স্বাস্থ্যকর্মী অন্য কোন হাসপাতালে যাওয়ার পরামর্শ (রেফারেল সিস্টেম) দিয়েছিল কিনা? (১= হ্যাঁ, ২= না)		
**505**	আপনার বা আপনার পরিবারের কোন সদস্যের দীর্ঘমেয়াদী কোন রোগ আছে কিনা? (১= হ্যাঁ, ২= না)		
**506**	যদি থাকে, তাহলে কোন ধরণের দীর্ঘমেয়াদী রোগ আছে? (একাধিক উত্তর গ্রহনযোগ্য)	১= উচ্চ রক্তচাপ, ২= ডায়াবেটিস, ৩= হৃদ রোগ, ৪= ক্যানসার, ৫= স্ট্রোক, ৬= হাঁপানি/ব্রংকাইটিস, ৭= কিডনি রোগ)	
**507**	দীর্ঘমেয়াদী রোগের জন্য কোন জায়গা থেকে স্বাস্থ্য সেবা নেয়া হয়েছিল? (একাধিক উত্তর গ্রহনযোগ্য)	১= সরকারী হাসপাতাল, ২= এনজিও পরিচালিত ক্লিনিক, ৩= প্রাইভেট ক্লিনিক, ৪= প্রাইভেট ডাক্তার, ৫= হোমিওপ্যাথিক ডাক্তার, ৬= স্থানীয় ফার্মেসী, ৭= অন্যান্য	
**508**	আপনার বা আপনার পরিবারের কোন সদস্যকে দীর্ঘমেয়াদী রোগের উন্নত চিকিৎসার জন্য কোন স্বাস্থ্যকর্মী অন্য কোন হাসপাতালে যাওয়ার পরামর্শ (রেফারেল সিস্টেম) দিয়েছিল কিনা? (১= হ্যাঁ, ২= না)		
**509**	আপনার এলাকা বা নিকটস্থ স্বাস্থ্যসেবা কেন্দ্রে প্রাথমিক এবং জরুরী চিকিৎসার ব্যবস্থা আছে কিনা? (১= হ্যাঁ, ২= না)		
**F. রোগ নির্নয় সংক্রান্ত সেবা (ডায়াগনষ্টিক সেবা সংক্রান্ত) ঃ**			
**601**	আপনার জানা মতে আপনার এলাকায় রোগ নির্নয় (রক্ত, কফ, মল, মূত্র ইত্যাদি পরীক্ষা) করার কোন ব্যবস্থা আছে কি? (১= হ্যাঁ, ২= না)		
**602**	আপনার এলাকায় কোথায় রোগ নির্নয় এর জন্য পরীক্ষা করা যায়? (একাধিক উত্তর গ্রহনযোগ্য)	১= সরকারী হাসপাতাল, ২= এনজিও পরিচালিত ক্লিনিক, ৩= প্রাইভেট ক্লিনিক/ডায়াগনস্টিক সেন্টার, ৪= অন্যান্য	
**G. পেপসেপ কর্মসূচী টেকসইকরণ**			
**701**	আপনি কি মনে করেন এই পেপসেপ প্রকল্পের স্বাস্থ্য সেবা আরোও কিছু দিন চালিয়ে যাওয়া উচিৎ? (১= হ্যাঁ, ২= না)		
**702**	পেপসেপ প্রকল্পের কর্মকান্ডে আপনি/আপনারা সন্তুষ্ট কি না? (১= হ্যাঁ, ২= না)		
**703**	পেপসেপ প্রকল্পের স্বাস্থ্য সেবা গ্রহন কালে কোন বাঁধা বা সমস্যার সম্মুখিন হয়েছেন কি না? (১= হ্যাঁ, ২= না)		
**704**	আপনি কি মনে করেন পেপসেপ প্রকল্প আপনার এলাকার লোকদের/জনগনকে সাহায্য করতে পেরেছে? (১= হ্যাঁ, ২= না)		

আপনাকে অনেক ধন্যবাদ
