# Supplementary material for: Water, sanitation, and hygiene (WASH) factors and the incidence of communicable diseases in Urban Bangladesh: Evidence from municipal areas
Source: PLoS Negl Trop Dis. 2025 Jul 31;19(7):e0013329. doi: 10.1371/journal.pntd.0013329 (PMC12312932; doi:10.1371/journal.pntd.0013329)
Supplement: Attachment — Submitted filename: PNTD-D-24-01678_Review comments.docx [file pntd.0013329.s005.docx]

Title: Urban Hygiene and the Incidence of Communicable Diseases: A Study of Bangladeshi Municipal Areas

**Abstract:**

Line 27-32: Better to add inclusion criteria of the participants.

Line 40-43: In conclusion, suggesting specific types of interventions, like community-based WASH programs or educational campaigns will add greater depth.

Line 44: WASH covers both sanitation and hygiene. Better to search for more suitable keywords instead of sanitation and hygiene. You can use the full form of WASH in keyword.

**Introduction:**

Line 58: “Bangladesh, with the eighth largest population globally.” Incomplete sentence.

Line 65: We can use ‘WASH condition’ instead of ‘WASH score’ for better clarity for normal audience.

Line 69: You mentioned the HIV prevalence rate is 1% of the entire population, which is very high. Need to cross-check and add one or two more citations to give this data a strong base.

Line 86-87: Kindly add some references of the research on communicable diseases and WASH practices conducted in Bangladesh and explain how your study will address existing gaps

Line 91: Mention the specific names of the two municipalities

**Method:**

Line 94-99: Need to mention briefly why Savar and Satkhira were chosen as study sites and how they represent the urban Bangladesh.

Line 106: Kindly add a separate line on inclusion criteria for better clarity and explain the reason for each point.

Line 115: You mentioned an established framework. It’s better if you add a reference so that readers can understand which framework was used.

Line 120-121: Provide examples of the minor adjustments made to improve clarity after pilot study.

Line 134: Need to explain what PEPSEP is.

**Result:**

Line 181: Mentioned figure 1 as this figure was described from line 181-186.

Line 181-186: Figure 1 showed 24.1% communicable disease. Here also showed the percentage of other communicable disease. Better to omit communicable disease percentage (24.1%) from this chart to avoid confusion. If you want to show the overall percentage, mention it in the figure headings.

**Discussion:**

Line 218-222: In this introductory part, if possible, it is better to keep focus on communicable disease according to the objective of the paper.

Line 227-228: This statement says that one-fourth of the participants have a communicable disease burden. How is this information reported? orally or by observing medical documents. Please include this information here. If orally taken, this can also be added in the limitation part.

Line 232: Why females are being affected more in this study population? Adding this explanation will increase the depth of your statement.

Line 239-240: Is the "Clean Care is Safer Care" campaign also conducted in Bangladesh? If yes, is there any update?

Line 246-247: Higher odds of developing chronic diseases or communicable diseases? In table 4 it showed against communicable diseases.

Line 270-279: It is not clear how this part related to the paper’s primary objective.

Line 284-285: As you mentioned, ‘it was impossible to compare the risk factors for the development of communicable diseases with regard to the socio-demographic context’; then how do you conduct the analysis in Table 4?

**Conclusion:**

Line 290-298: It is better to mention how the study's findings will help stakeholders mitigate the risk.
