# Supplementary material for: Water, sanitation, and hygiene (WASH) factors and the incidence of communicable diseases in Urban Bangladesh: Evidence from municipal areas
Source: PLoS Negl Trop Dis. 2025 Jul 31;19(7):e0013329. doi: 10.1371/journal.pntd.0013329 (PMC12312932; doi:10.1371/journal.pntd.0013329)
Supplement: Attachment — Submitted filename: Reviewers comments.rtf [file pntd.0013329.s006.rtf]

Reviewers comments
1. In the problem statement, It is only Bugoma mentioned to have STH, why was Kilifi left out considering it is still prevalent for STH.
2. What was the justification of having the two sites?
3. What was the justification of having urban and periurban study sites?
4. The timeline can be revised to include specific activities.


Verdict: Can proceed to the next level.
