# Supplementary material for: Water, sanitation, and hygiene (WASH) factors and the incidence of communicable diseases in Urban Bangladesh: Evidence from municipal areas
Source: PLoS Negl Trop Dis. 2025 Jul 31;19(7):e0013329. doi: 10.1371/journal.pntd.0013329 (PMC12312932; doi:10.1371/journal.pntd.0013329)
Supplement: Attachment — Submitted filename: Reviewers_Comments.docx [file pntd.0013329.s008.docx]

| **Reviewer** | **Reviewer Comment** | **Author Response** |
| --- | --- | --- |
| **Reviewer 1** | Abstract: Line 27–32: Better to add inclusion criteria of the participants. | The abstract has been updated to mention the inclusion criteria: adults aged 18 and above from poor and marginalised communities in Savar and Satkhira municipalities. |
| Reviewer 1 | Abstract: Line 40–43: In conclusion, suggesting specific types of interventions, like community-based WASH programs or educational campaigns will add greater depth. | Specific interventions such as community WASH campaigns and education programs have been added to the conclusion section for practical relevance. |
| Reviewer 1 | Abstract: Line 44: WASH covers both sanitation and hygiene. Better to search for more suitable keywords instead of sanitation and hygiene. You can use the full form of WASH in keyword. | Keywords updated to include the full form “Water, Sanitation, and Hygiene (WASH)” to reflect integrated terminology. |
| Reviewer 1 | Introduction: Line 58: “Bangladesh, with the eighth largest population globally.” Incomplete sentence. | Sentence revised for clarity: “Bangladesh, with the eighth largest population globally, continues to face considerable WASH-related public health challenges.” |
| Reviewer 1 | Introduction: Line 65: We can use ‘WASH condition’ instead of ‘WASH score’ for better clarity for normal audience. | Replaced “WASH score” with “WASH condition” for improved clarity. |
| Reviewer 1 | Introduction: Line 69: You mentioned the HIV prevalence rate is 1% of the entire population, which is very high. Need to cross-check and add one or two more citations to give this data a strong base. | The HIV prevalence has been corrected to less than 0.01% with updated references from Bangladesh Government Health Statistics. |
| Reviewer 1 | Introduction: Line 86–87: Kindly add some references of the research on communicable diseases and WASH practices conducted in Bangladesh and explain how your study will address existing gaps. | Added references to relevant studies and clearly stated that our urban-focused approach fills the gap in prior rural/intervention-based research. |
| Reviewer 1 | Introduction: Line 91: Mention the specific names of the two municipalities. | The names “Savar” and “Satkhira” are now explicitly included in the manuscript. |
| Reviewer 1 | Method: Line 94–99: Need to mention briefly why Savar and Satkhira were chosen as study sites and how they represent the urban Bangladesh. | Added rationale stating Savar represents peri-urban industrial zones and Satkhira represents underdeveloped semi-urban contexts, providing diversity in urban representation. |
| Reviewer 1 | Method: Line 106: Kindly add a separate line on inclusion criteria for better clarity and explain the reason for each point. | A dedicated section on inclusion criteria was added: adults ≥18 years, resident of identified communities, willing to consent. These criteria ensured ethical, informed, and context-relevant participation. |
| Reviewer 1 | Method: Line 115: You mentioned an established framework. It’s better if you add a reference so that readers can understand which framework was used. | Reference to Joint Monitoring Programme (JMP) for WASH indicators has been added. |
| Reviewer 1 | Method: Line 120–121: Provide examples of the minor adjustments made to improve clarity after pilot study. | Clarified that changes included simplifying technical terms for local comprehension and clarifying frequency terms related to hand hygiene. |
| Reviewer 1 | Method: Line 134: Need to explain what PEPSEP is. | PEPSEP is now explained as the “Health and Nutrition Voucher Scheme for Poor, Extreme Poor, and Socially Excluded People” to improve healthcare access. |
| Reviewer 1 | Result: Line 181: Mentioned figure 1 as this figure was described from line 181–186. | Figure 1 is now explicitly referenced in the related paragraph. |
| Reviewer 1 | Result: Line 181–186: Figure 1 showed 24.1% communicable disease. Here also showed the percentage of other communicable disease. Better to omit communicable disease percentage (24.1%) from this chart to avoid confusion. If you want to show the overall percentage, mention it in the figure headings. | Modified figure and caption to move the 24.1% to the title and avoid confusion in bar representation. |
| Reviewer 1 | Discussion: Line 218–222: In this introductory part, if possible, it is better to keep focus on communicable disease according to the objective of the paper. | Revised the paragraph to emphasise communicable disease burden in line with study objectives. |
| Reviewer 1 | Discussion: Line 227–228: This statement says that one-fourth of the participants have a communicable disease burden. How is this information reported? Orally or by observing medical documents. Please include this information here. If orally taken, this can also be added in the limitation part. | Clarified that all disease information was self-reported during interviews, and this limitation is now mentioned in the discussion. |
| Reviewer 1 | Discussion: Line 232: Why females are being affected more in this study population? Adding this explanation will increase the depth of your statement. | Added rationale: Women’s domestic roles involve higher exposure to water and sanitation sources, which may explain their greater disease burden. |
| Reviewer 1 | Discussion: Line 239–240: Is the “Clean Care is Safer Care” campaign also conducted in Bangladesh? If yes, is there any update? | Yes, Bangladesh participated in the 2007 WHO regional workshop and the campaign was adopted in national infection control strategies. This section is now moved to introduction. |
| Reviewer 1 | Discussion: Line 246–247: Higher odds of developing chronic diseases or communicable diseases? In table 4 it showed against communicable diseases. | Clarified this statement now specifically refers to communicable diseases, as shown in Table 4. |
| Reviewer 1 | Discussion: Line 270–279: It is not clear how this part related to the paper’s primary objective. | Revised paragraph to clearly relate chronic illness with communicable disease vulnerability, consistent with study objectives. |
| Reviewer 1 | Discussion: Line 284–285: As you mentioned, ‘it was impossible to compare the risk factors for the development of communicable diseases with regard to the socio-demographic context’; then how do you conduct the analysis in Table 4? | Clarified that despite sample homogeneity, sufficient variation existed across key variables to conduct subgroup analysis. However, it limits the depth of subgroup analysis, which is acknowledged in the limitations. |
| Reviewer 1 | Conclusion: Line 290–298: It is better to mention how the study’s findings will help stakeholders mitigate the risk. | Conclusion now includes specific implications for policymakers and health stakeholders regarding targeted urban WASH interventions and chronic illness management. |
| **Reviewer** | **Reviewer Comment** | **Author Response** |
| Reviewer 2 | A description of the study sites and why they were selected would be useful. | Added rationale stating Savar represents peri-urban industrial zones and Satkhira represents underdeveloped semi-urban contexts, providing diversity in urban representation. |
| Reviewer 2 | L. 101-102: further details should be provided on the sampling procedure in order to clarify how "poor, extremely poor, marginalised and socially excluded" individuals were identified to participate in the survey and how households were located by the survey teams. The definition of these categories should also be clarified (what are income levels of "poor" and "extremely poor" households? Providing this information would help contextualise findings in the Results section). | Participants were selected using a purposive sampling method guided by local data. Based on urban living costs and average household size, households earning ≤ BDT 10,000/month were defined as extremely poor, and those earning BDT 10,001–20,000/month as poor. |
| Reviewer 2 | Questionnaires should be provided as supplementary information together with the manuscript - and anonymised data. | The questionnaire has been added as supplementary material as per the recommendation. And dataset is available from corresponding author based on reasonable request. |
| Reviewer 2 | L. 130: what is meant by household type? What about household size? who were the preferred/targeted interviewees within each household (e.g. head of household)? | 'Household type' was clarified as the structural nature of the residence (e.g., tin shed, semi-pucca). The preferred respondent was the adult head of household or a knowledgeable adult family member. |
| Reviewer 2 | L. 131: consider replacing with "WASH practices", and indicate whether access and/or practices were assessed. | Replaced “hygiene-related practices” with “WASH practices” and clarified that both access (e.g., type of toilet, water source) and behaviors (e.g., handwashing practices) were assessed. |
| Reviewer 2 | L. 133-134: a recall period of 1 year is long and prone to bias; please provide a justification for this. The relevance of chronic diseases in relation to WASH should be clarified. The abbreviation "PEPSEP" needs to be defined and additional background provided. | Justified 1-year recall period as necessary to capture seasonal trends and infrequent disease episodes. Chronic diseases were included due to their influence on susceptibility to infections. PEPSEP is defined and contextualized in the methods. |
| Reviewer 2 | The obtention of written consent is mentioned several times. | We have reviewed and streamlined all references to written consent for clarity and conciseness, retaining only essential mention. |
| Reviewer 2 | L. 139: "confidence" or "confidentiality"? | Corrected “confidence” to “confidentiality” to align with ethical terminology. |
| Reviewer 2 | L. 140-146: the rationale for using both Chi-square tests and a logistic regression is unclear. The analysis should focus on answering the research questions defined in the manuscript. The logistic regression alone might be more appropriate than repeating Chi-square tests for each variable; the confounders included in the analysis should be clearly stated, and a rationale for selecting variables to be included in the model should be given (including consideration of potential collinearity between variables). | Clarified that chi-square tests were used for univariate associations and logistic regression for multivariable analysis. Confounders were chosen based on theoretical plausibility, previous literature, and statistical significance. Multicollinearity was assessed and confirmed acceptable via VIF scores. |
| Reviewer 2 | It would be useful to align WASH terminology with the definitions of water and sanitation used for Sustainable Development Goal #6 (WHO/UNICEF Joint Monitoring Programmes) and/or explain how WASH solutions and terms that might be used locally correspond to the WASH ladders. | Revised text now aligns definitions of “sanitary latrines” and “improved water sources” with the JMP’s WASH ladders. A clarifying sentence was added to explain how local terms relate to global definitions. |
| Reviewer 2 | L. 161-162: "supply water" is unclear - is this referring to piped water? | Rephrased to specify “piped water supplied by municipal sources.” |
| Reviewer 2 | L. 163-164: "sanitary toilets" is unclear | Clarified “sanitary toilets” as latrines that hygienically separate human excreta from human contact, consistent with WHO/UNICEF definitions. |
| Reviewer 2 | For the health-related results (L. 168-170), clarity is needed regarding the time period considered (is it the 1 year referenced in the methods?). It should also be clear whether the disease affected any member of the household or only the survey respondent. | Clarified that reported diseases covered the past 12 months and referred to any household member, as reported by the head/respondent. |
| Reviewer 2 | Table 3: if kept, report the p-value as smaller than a threshold rather than zero. | Revised Table 3 p-values to be reported as “<0.001” instead of “0.000.” |
| Reviewer 2 | The discussion includes information that might be better placed in introduction and fails to provide clear recommendations for future WASH interventions. | Discussion has been revised to shift background material to the introduction and now includes specific, actionable recommendations like promoting piped water systems and educational campaigns. |
| Reviewer 2 | Linking findings to the WASH'Benefits trial, a major intervention study which assessed WASH interventions sin Bangladesh, would be useful, too. | The discussion now references the WASH Benefits trial and contrasts its intervention-based rural focus with our observational urban findings. |
| Reviewer 2 | Limitations related to the assessment of diseases burden based on self-report should be acknowledged. | This limitation is now explicitly discussed in the manuscript's limitations section. |
| Reviewer 2 | L. 266-267: this does not seem to be supported by the survey data (cross-sectional). | Sentence revised to clarify that only associations (not causation) are inferred due to the cross-sectional nature of the study. |
| Reviewer 2 | L. 286-288: the statement should be revised - the study design did not allow to establish causal relationships. | The manuscript has been revised to remove any implication of causal inference and explicitly states the limitations of the study design. |
| Reviewer 2 | The introduction can be streamlined to focus on the burden of WASH-preventable diseases such as diarrhoeal diseases and lower respiratory infections (e.g. using the Global Burden of Disease studies and national statistics). It should also include a clear description of WASH access in Bangladesh (% access to different services, differences urban/rural if relevant - where are the most important gaps). | The introduction was restructured for flow and emphasis on WASH-preventable disease burden with added national statistics on WASH access and urban-rural differences. |
| Reviewer 2 | The title only mentions hygiene but the scope of the survey covers water, sanitation and hygiene - consider adapting accordingly. | Title revised to reflect the full scope: “Water, Sanitation, and Hygiene (WASH) Factors and the incidence of Communicable Diseases in Urban Bangladesh: Evidence from Municipal Areas.” |
| Reviewer 2 | Wikipedia (ref. 7) should preferably not be included among references. | Wikipedia citation removed and replaced with an appropriate source. |
| Reviewer 2 | The manuscript reports data from a cross-sectional survey of >600 households assessing disease burden and WASH access in two communities in Bangladesh. The study could potentially help identify risk factors for infectious diseases in vulnerable populations. However, I have concerns about (i) the disease burden assessment based on self-report, with a recall period of 1 year, which is likely unreliable; (ii) the statistical analyses, using both (repeated) Chi-square tests and logistic regression, with a lack of clarity regarding the tested hypotheses; (iii) the discussion, which fails to provide clear recommendations based on the survey findings. | We appreciate the concerns and have addressed each one: (i) we clarified disease data were self-reported and acknowledged the limitation; (ii) we clarified the rationale for using both chi-square and logistic regression and explained variable selection; (iii) we revised the discussion to clearly present WASH-focused recommendations based on findings. |
